# Scientific misconduct and medical publishing

**DOI:** 10.4103/1817-1737.36547

**Published:** 2007

**Authors:** Yaseen M. Arabi

**Affiliations:** *Intensive Care Department, College of Medicine, King Saud Bin Abdulaziz University, King Abdulaziz Medical City, KSA*

It is a basic understanding that ethics of medical research should be observed throughout the research process, from the conception of the study idea to the conduction of the study and interpretation the results to writing the manuscript. Unfortunately, the integrity of research ethics is often violated. Violations vary in seriousness but all represent deviation from the honesty and originality expected from investigators. Furthermore, these practices make the scientific merit of the involved manuscripts suspect. Several forms of research misconduct have been encountered including plagiarism, falsification, fabrication, authorship fraud, and conflict of interest. Plagiarism refers to copying any of the writings of previous publications without attribution. This may include copying sentences, paragraphs, or whole articles.[1,2] Self- plagiarism, which is using own writing from a previous publication, without permission.[3] Duplicate publication is a form of self- plagiarism. Many cases of plagiarism are detected at various stages of the peer review process. However, this mechanism of detection is far from being perfect, and many other cases escape detection.[4] However, more cases are being detected after publication via searching internet engines, such as Google.[5] Additionally, software programs have been developed to enable journals to spot plagiarism.[6] As medical journals thrive to present original and truthful literature, there is no room for accepting misconduct and fraud. Such practices are not only unethical but may be illegal in certain countries like the United States.[1] Such cases of research misconduct should serve as the impetus for the medical community to develop guidelines and procedures to guard against undesirable behaviors. Journals should undertake swift and firm measures in order to eliminate and prevent such practices. Once misconduct is identified in a publication, the journal should condemn such an act in the next issue and request the author to retract his/her paper.[7] The journal then should publish a retraction and provide and electronic link to the fraudulent article's citation. Another duty of the scientific community is to verify the integrity of other articles published by the author of a fraudulent article. The journal should notify the authors' institution to investigate the incident and take the necessary action. Journals should work in a network to notify each other when a fraudulent article is identified so it will not be published somewhere else. Journals must not publish any article with citation of fraudulent article. Finally, there is a need for a governmental agency that has the authority to investigate and punish guilty scientists. An example is the Office of Research Integrity (ORI) in the United States.[8] The Annals of thoracic Medicine is committed to preventing any form of research misconduct and will take the necessary actions against violators.
